# Repetitive invasive lung function maneuvers do not accentuate experimental fibrosis in mice

**DOI:** 10.1038/s41598-024-64548-w

**Published:** 2024-06-14

**Authors:** Tina Röpke, Franziska Aschenbrenner, Lars Knudsen, Tobias Welte, Martin Kolb, Ulrich A. Maus

**Affiliations:** 1 Division of Experimental Pneumology, Hannover Medical School, Hannover, Lower Saxony Germany; 2 Institute of Functional and Applied Anatomy, Hannover Medical School, Hannover, Lower Saxony Germany; 3 Clinic for Pneumology, Hannover Medical School, Hannover, Lower Saxony Germany; 4 Department of Medicine, Pathology, and Molecular Medicine, McMaster University, Hamilton, Ontario Canada; 5 German Center for Lung Research, Partner Site BREATH, Hannover, Lower Saxony Germany

**Keywords:** Animal disease models, Respiratory tract diseases

## Abstract

Assessment of lung function is an important clinical tool for the diagnosis and monitoring of chronic lung diseases, including idiopathic pulmonary fibrosis (IPF). In mice, lung function maneuvers use algorithm-based ventilation strategies including forced oscillation technique (FOT), negative pressure-driven forced expiratory (NPFE) and pressure–volume (PV) maneuvers via the FlexiVent system. This lung function test (LFT) is usually performed as end-point measurement only, requiring several mice for each time point to be analyzed. Repetitive lung function maneuvers would allow monitoring of a disease process within the same individual while reducing the numbers of laboratory animals. However, its feasibility in mice and impact on developing lung fibrosis has not been studied so far. Using orotracheal cannulation without surgical exposure of the trachea, we examined the tolerability to repetitive lung function maneuvers (up to four times) in one and the same mouse, both under healthy conditions and in a model of AdTGF-β1 induced lung fibrosis. In essence, we found that repetitive invasive lung function maneuvers were well tolerated and did not accentuate experimental lung fibrosis in mice. This study contributes to the 3R principle aiming to reduce the numbers of experimental animals used in biomedical research.

## Introduction

Idiopathic pulmonary fibrosis (IPF) is the most severe form of interstitial lung diseases with unknown cause and characterized by continuous deposition of extracellular matrix components eventually leading to an irreversible loss of lung function^1–3^. Assessment of lung function is instrumental to diagnosis and monitoring of chronic lung diseases. A strong predictor of mortality in IPF patients is a decline in forced vital capacity (FVC), with reduced survival of those patients displaying a 10% drop in FVC within 6 months^2,4–7^. Static lung compliance also decreases in early IPF, thus further underscoring the importance of lung function tests (LFTs) in the management of interstitial lung diseases^1,8–10^.

Experimental studies in mice play an important role in lung fibrogenesis research^11^. Assessment of lung function in mice can be achieved via noninvasive or invasive techniques. Noninvasive techniques in spontaneously breathing mice are imprecise due to upper airway resistance and bronchoconstriction^12,13^. For example, body plethysmography allows repetitive noninvasive lung function measurements in rodents but the reproducibility and precision of this method is limited^14,15^. In contrast, the FlexiVent system allows invasive measurement of lung function in mice via different maneuvers under accurately controlled experimental conditions, thereby reaching high sensitivity and reproducibility. However, this technique is usually (but not always) performed as endpoint measurement requiring a surgical intervention. Assessment of lung function over time would therefore require multiple measurements in different animals, resulting in exceedingly high numbers of mice for a lung function study, depending on the number of time points and the strength of the effect under study. Further, experimental model systems would better reflect the clinical situation when longitudinal assessment of lung function is performed within one and the same mouse with minimal experimental manipulation.

Previous studies showed that repetitive LFT via orotracheal intubation is feasible in healthy mice^16,17^. However, whether or not repetitive LFT might additionally worsen experimental lung fibrosis has not been studied yet. In the current study, we explored the effect of repeated LFT on developing AdTGF-β1 induced lung fibrosis within the same mice.

## Materials and methods

### Animals

Adult female C57BL/6J mice (10 weeks old, weight 20–24 g) were purchased from Janvier Labs (Le Genest-Saint-Isle, France). All procedures were in accordance with the European Guidelines for Animal Experiments (Directive 2010/63/EU), the German Animal Welfare Law and the ARRIVE guidelines^18^. The study protocol was approved by the local Institutional Animal Care and Research Advisory Committee and permitted by the Lower Saxony State Office for Consumer Protection and Food Safety (Permission Number 21/3823).

### Treatment of mice

Mice were anaesthetized by intraperitoneal (i.p.) injection of 75 mg/kg body weight (b.w.) ketamine hydrochloride (Anesketin, Albrecht, Aulendorf, Germany) and 10 mg/kg b.w. xylazine hydrochloride (Rompun, Bayer, Leverkusen, Germany). Mice were monitored during anesthesia and were inspected and weighed every day during the course of the experiment. General welfare conditions of the mice were classified by a scoring system for the occurrence of disease symptoms (including breath rate, activity, weight loss, ruffled fur and self-isolation) that was approved by our institutional ethics committee and our governmental authorities.

For intratracheal instillation of vectors, we inserted an Abbocath-T catheter (26G; Hospira Venisystems, Lake Forest, USA) under visual control into the trachea of mice. Adenoviral vector carrying the cDNA for biologically active porcine TGF-β1 (AdTGF-β1) or empty control vector (AdCL) were prepared as previously described and were applied orotracheally (o.t.) at 10^8^ plaque-forming units (PFU) in 50 µl PBS per mouse^19^. Lung fibrosis in response to AdTGF-β1 is typically established in mice by day 14 and remains stable until day 28 with a decline thereafter^19^. Therefore, effects of repeated LFT on lung fibrosis were examined starting by day 14 until day 28 post-treatment. Lung function of mice with experimental lung fibrosis was defined based on assessment of inspiratory capacity (IC), lung tissue elastance H, and static compliance Cst. In addition, histopathological features of lung fibrosis (widened alveolar septa, increased lung interstitial collagen deposition) and biochemical parameters such as lung hydroxyproline contents were collected from AdTGF-β1 exposed mouse lungs to define the degree of experimental lung fibrosis.

### Experimental groups

Mice were divided in two experimental groups. Thirty-six animals were used for the 1 × LFT group as well as for the 4 × LFT group, and these mice were all analyzed on day 29. In experimental group 1, mice underwent a single LFT at day 28 post AdCL or AdTGF-β1 treatment (1 × LFT). In experimental group 2, basal lung function of mice was determined on day 0, followed by immediate administration of AdCL or AdTGF-β1, and repeated assessment of lung function on days 14, 21 and 28 (4 × LFT). In selected experiments, we also determined the effect of 2 × LFT (performed on days 0 and 14 post-vector application) on BAL leukocyte subsets and lung histopathology. To control for effects caused by repeated anesthesia only, mice of group 1 were anaesthetized at the same time points (i.e., at days 14, 21 and 28) as mice of group 2. After treatment, mice were brought back to their cages with free access to food and water and were monitored for the development of disease symptoms daily. At day 29, bronchoalveolar lavage was carried out on all mice that were used for FACS analysis of leukocyte subsets in BAL fluids. Subsequently, lungs were harvested and additionally used for determination of hydroxyproline contents or flow cytometry analyses of lung leukocyte subsets, or lung histopathology, unless otherwise stated.

### Assessment of lung function

We performed repeated LFT in mice on days 0, 14, 21 and 28 post-adenoviral application. To this end, anaesthetized mice were intubated with a sterile blunt 19G cannula followed by connection to a flexiVent respirator (SCIREQ, Montreal, Canada). 19G catheters were employed based on the finding that these catheters caused no injury to the trachea during intubation. The flexiVent system was calibrated before each LFT so that mechanical properties of the flexiVent system and the employed tracheal cannula was taken into consideration for the calculations of lung mechanical properties. Ventilation was performed with a tidal volume of 10 ml/kg body weight, a respiratory rate of 150/min, and at a positive end-expiratory pressure (PEEP) of 3 cm H_2_O^20–22^. Two recruitment maneuvers (deep lung inflation, 30 cm H_2_O) as well as three pressure-controlled quasi-static pressure–volume (PV) loops were applied. We used the forced oscillation technique (FOT) involving baseline ventilation for 5 min with 30 s intervals of low frequency perturbations.

The inspiratory capacity (IC) reflecting the amount of air to be inhaled after the end of a normal expiration was calculated during deep inflation. The deep inflation maneuver facilitates the opening of closed lung areas, and restores airway patency and normalizes lung volume. Tissue elastance (H) reflects the lung resistance to distention when mechanical load is applied, and was calculated by fitting the constant-phase model to impedance spectra obtained during FOT. Therefore, tissue elastance is an indicator of lung stiffness and usually increases during developing lung fibrosis. Quasi-static compliance (Cst) reflecting the distensibility of the lung was determined by the mean of the three PV loop values using Salazar-Knowles equation^20,21^.

### Bronchoalveolar lavage

Bronchoalveolar lavage of mice was performed as previously described in detail^23^. Quantification of BAL fluid macrophage subsets was done on Pappenheim-stained cytocentrifuge preparations. Tissue-resident alveolar macrophages (rAM) with marginal, round nuclei, a light-blueish cytoplasm and strong vacuolization were distinguished from monocyte-derived exudate macrophages (ExMacs) with kidney-shaped nuclei, few vacuolization and mid-blueish cytoplasm. Neutrophils were identified based on their typical polymorphonuclear appearance along with a slightly red-stained cytoplasm in HE-stained cytospin preparations.

### Hydroxyproline assay

Total lung collagen contents were determined by using hydroxyproline assays as described previously^19,24^. Briefly, whole lungs were removed from experimental animals and homogenized in 500 µl PBS. We then added 500 µl of 12 N HCl to samples and hydrolyzed them at 120 °C for 6 h. Afterwards, 5 µl of each sample was mixed with 5 µl citrate/acetate buffer (0.24 M citric acid, 0.88 M sodium acetate, 0.21 M acetic acid, 0.85 M sodium hydroxide solved in 100 ml A. dest) in a 96-well plate. 100 µl of chloramine T solution was added and samples were incubated for 30 min at room temperature. Next, we added 100 µl DMBA (4-(Dimethylaminobenzaldehyde) to the samples and incubated them for 30 min at 37 °C. The absorbance of each sample was measured at 550 nm (reference wavelength 650/655 nm). Hydroxyproline levels of the lungs were determined by referring to a standard curve of purified trans-4-Hydroxy-l-Prolin (Merck, Darmstadt, Germany) samples that were assayed in parallel.

### Lung histology

Lung histology was performed as described recently^22^. In brief, whole lungs of mice were inflated in situ with formaldehyde solution (4%, Histofix; Roth, Karlsruhe, Germany), followed by removal of the lungs and immersion in formaldehyde solution for at least 24 h at room temperature. Paraffin-embedded lung tissue sections (3 µm) were stained with hematoxylin and eosin (H&E) or Elastica van Gieson (EvG) followed by histopathological examination of sections using an Olympus BX-53 microscope (Olympus, Tokio, Japan) at × 10 original magnification.

### Immunophenotypic analysis of lung leukocytes

After collection of bronchoalveolar lavage fluids, lungs were removed, cut into small pieces and digested in RPMI1640 supplemented with collagenase A (5 mg/ml) and DNase I (1 mg/ml). CD45^+^ leukocytes were enriched via magnetic cell separation (MACS Purification Kit; Miltenyi Biotec). A BD LSR Fortessa (BD Biosciences) equipped with a four-laser system was used for flow cytometric analysis. Lymphocytes were gated according to their forward scatter area (FSC-A)/side scatter area (SSC-A) and CD45 cell surface expression. B cells were identified based on their CD19 cell surface expression, while T cell subsets were gated according to their CD45 + /CD3 + expression and were further sub-gated according to their CD4 + or CD8 + antigen expression. Macrophages were gated according to their FSC-A/SSC-A and their green autofluorescence properties and their CD45 and F4/80 cell surface antigen expression. Subsequently, macrophages were further characterized as CD11c + , CD11b- resident alveolar macrophages, and CD11c + , CD11b + exudate macrophages. Neutrophils were identified according to their FSC-A/SSC-A characteristics and Ly6G cell surface expression. Post-acquisition compensation settings and data processing were performed using BD FACS Diva software.

### Statistical analysis

Statistical significance between groups was assumed when *p* values were < 0.05. Differences between experimental groups were analyzed by one-way ANOVA with Tukey's multiple comparisons test for normally distributed data or Mann–Whitney *U* test using GraphPad Prism software (version 8.0). To test for normal data distribution, we created a QQ plot in Graph pad prism (version 8.0) employing Shapiro–Wilk normality test. Sample sizes were calculated by G power version 3.1.9.7 based on available data from previous studies on lung collagen contents of mice with established lung fibrosis.

## Results

### Repeated lung function measurement does not cause weight loss or inflammation in mice

**Figure 1:**
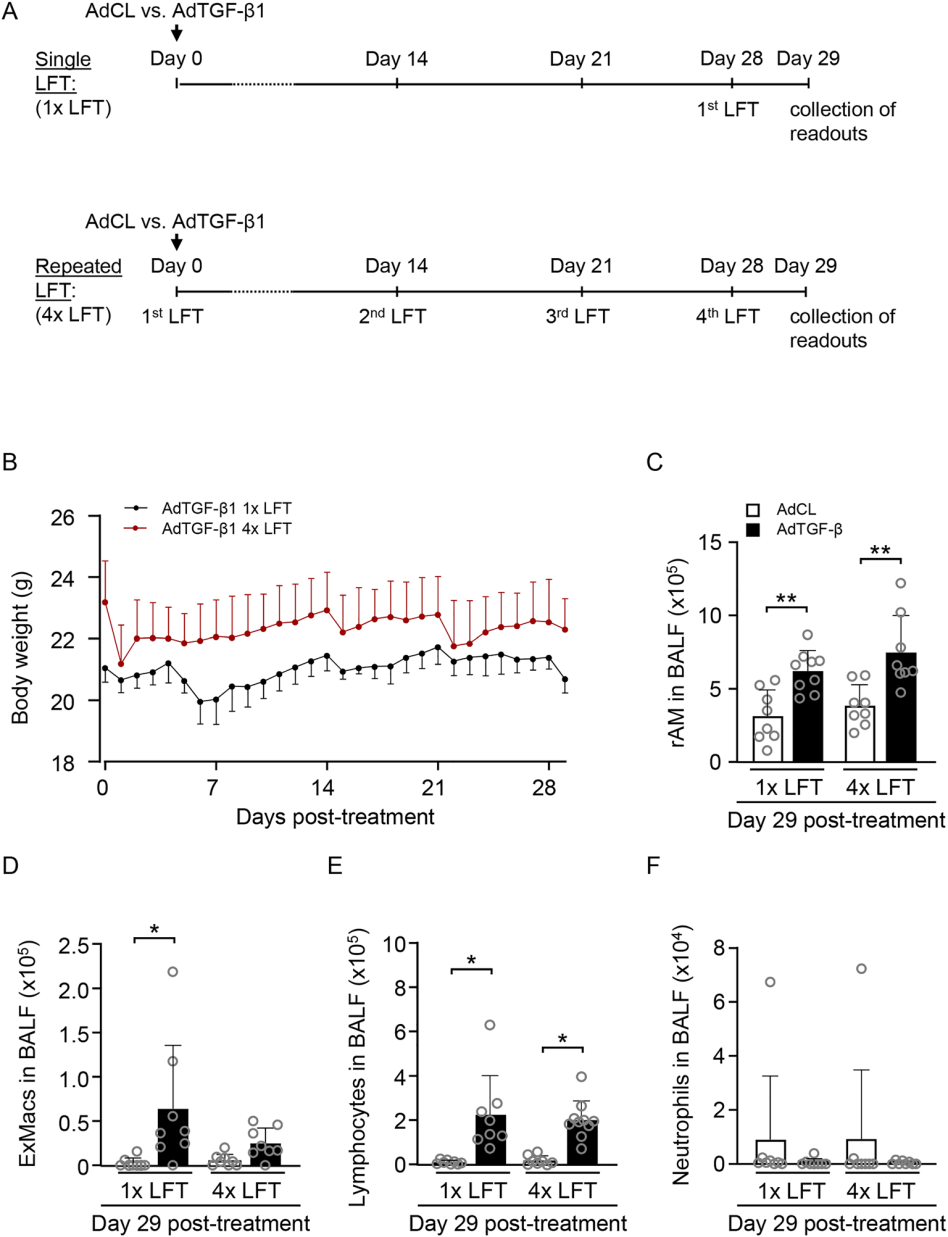
Repeated lung function measurement does not cause weight loss or inflammation in mice. (**A**) Experimental setup. Mice of the single lung function (1 × LFT) group were exposed to AdCL or AdTGF-β1 (1 × 10^8^ PFU/mouse) and received additional anesthesia at days 14 and 21. On day 28, mice underwent LFT and were analyzed on day 29 post-treatment. Mice in the 4 × LFT group underwent lung function tests at day 0 followed by administration of AdCL or AdTGF-β1 (1 × 10^8^ PFU/mouse). Lung function was measured at days 14, 21 and 28 followed by endpoint analysis at day 29. (**B**) Body weight of AdTGF-β1 treated mice with one LFT at day 28 (black line) or four LFT (red line). (**C**–**F**) Resident alveolar macrophages (rAM; **C**), exudate macrophages (ExMacs; **D**), lymphocytes (**E**) and neutrophils (**F**) in BAL fluid of mice exposed to AdCL (white bars) or AdTGF-β1 (black bars) after one or four LFT, as indicated. Data are presented as mean ± SD of n = 8 mice per group and time point and are representative of two independent experiments. *p < 0.05, **p < 0.01 compared to disease control (Mann–Whitney U test).

In the first set of experiments, we examined the effect of a single versus repeated LFT on weight loss and distribution of BAL fluid cellular constituents in mice with or without experimental fibrosis (Fig. 1A). Overall, mice tolerated repeated LFT without adverse effects on well-being. In AdTGF-β1 treated mice subjected to 4 × LFT, a slight and non-significant drop in body weight was observed after each LFT, which was also seen in mice of the 1 × LFT group after anesthesia on days 0, 14 and 21, suggesting anesthesia- rather than LFT-related effects on body weight (Fig. 1B).

Analysis of BAL cells at 24 h after the final LFT revealed only minor and non-significant differences between groups receiving single or repeated LFT. More specifically, in both groups (1 × vs. 4 × LFT), numbers of resident alveolar macrophages (rAM), exudate macrophages (ExMacs) and lymphocytes increased significantly in AdTGF-β1 as compared to AdCL treated mice, however with no apparent difference between groups at day 15 or 29 (Fig. 1C–E, Supplementary Fig. S1). Of note, repeated lung function measurement in mice with or without lung fibrosis did not trigger neutrophil influx into the lungs of mice serving as a sensitive marker of early lung inflammation (Fig. 1F).

### Repeated analysis of lung function does not alter lung mechanics in mice with or without lung fibrosis

**Figure 2:**
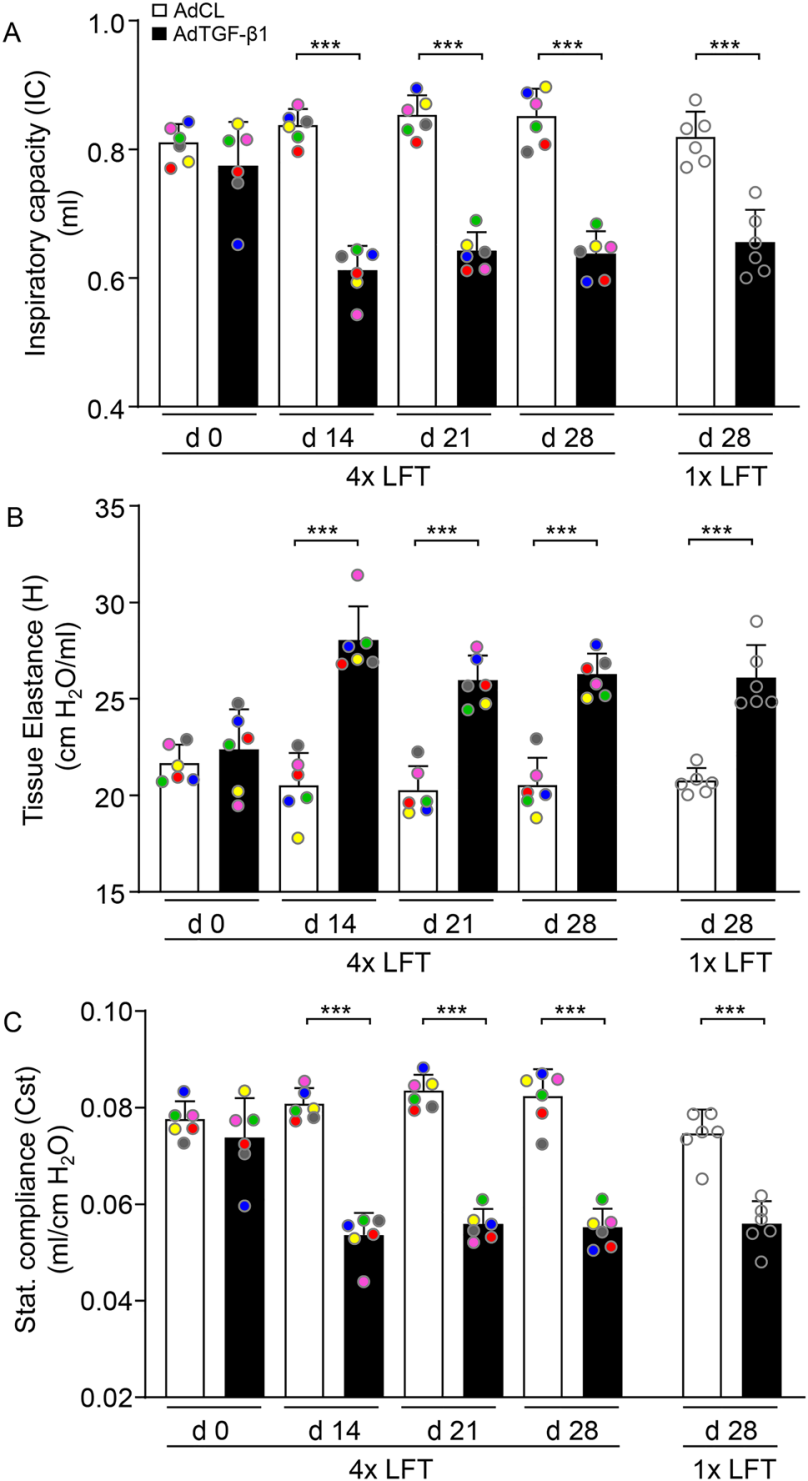
Repeated analysis of lung function does not alter lung mechanics in mice with or without lung fibrosis. (**A**–**C**) Inspiratory capacity (IC; **A**), tissue elastance (H; **B**) and static compliance (Cst; **C**) in mice exposed to AdCL (white bars) or AdTGF-β1 (black bars) after one or four LFTs, as indicated. Each color represents a single mouse in the designated group during four LFTs. Data are presented as mean ± SD of n = 6 mice per group and time point and are representative of two independent experiments. **p < 0.01, ***p < 0.001 compared to disease control (One-way ANOVA).

We next determined the effect of single as compared to repeated LFT on inspiratory capacity, tissue elastance and static compliance in individual mice either exposed to AdCL or AdTGF-β1 (Fig. 2). Mice exposed to AdCL and then subjected to repeated LFT demonstrated a normal IC over time (Fig. 2A). In contrast, mice exposed to AdTGF-β1 and then subjected to repeated LFT showed a significant decline in IC on days 14, 21 and 28, which however, was not different compared to AdTGF-β1 exposed mice subjected to one LFT only (Fig. 2A).

We next assessed lung tissue elastance (H) in AdCL and AdTGF-β1 exposed mice after one or four LFT. AdCL treated mice did not show changes in tissue elastance, whether or not exposed to four LFT. As expected, lung tissue elastance increased in mice exposed to AdTGF-β1, as assessed by single LFT on day 28. Mice of the 4 × LFT group showed a significantly increased tissue elastance on days 14, 21 and 28 post AdTGF-β1 compared to AdCL, which however, was not significantly different compared to tissue elastance observed in AdTGF-β1 exposed mice subjected to single LFT on day 28 post-treatment (Fig. 2B).

Finally, 4 × LFT did not alter static compliance (Cst) values in AdCL exposed mice, while AdTGF-β1 exposure led to a decreased Cst in mice of the repeated LFT group on days 14, 21 and 28 relative to AdCL. Again, no significant difference in Cst values was observed between AdTGF-β1 exposed mice subjected to single as compared to repeated LFT (Fig. 2C).

### Repeated lung function maneuvers do not affect developing lung fibrosis in mice

**Figure 3:**
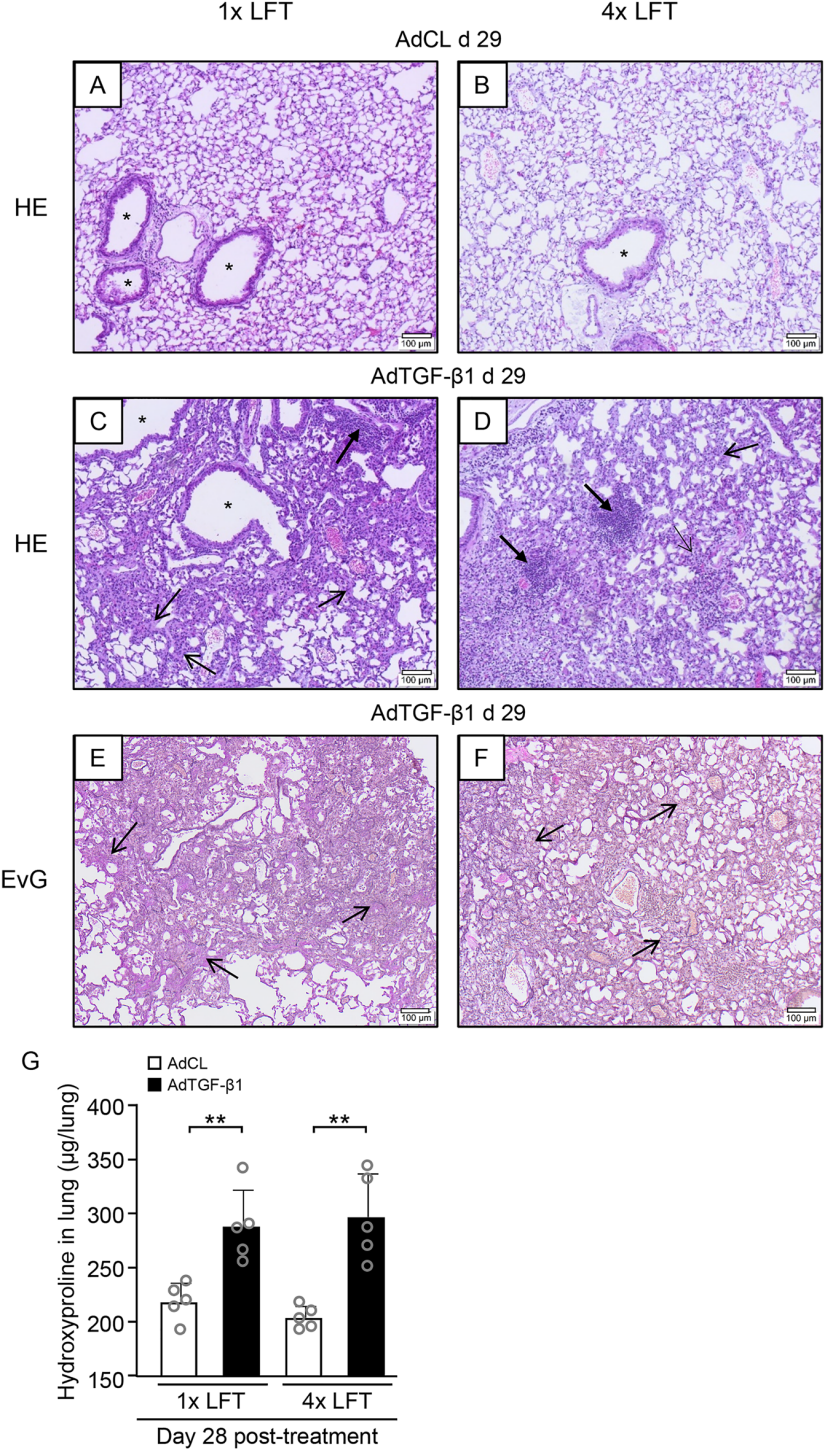
Repeated lung function maneuvers do not affect developing lung fibrosis in mice. (**A**–**F**) Histopathology of mice exposed to AdCL (**A**), or AdTGF-β1 (**C**, **E**) with one LFT at day 28 or mice exposed to AdCL (**B**), or AdTGF-β1 (**D**, **F**) with four LFT at day 0, 14, 21 and 28. Lung sections were stained with hematoxylin/eosin (HE) (**A–D**) or Elastica van Gieson (EvG) (**E**, **F**) for assessment of lung tissue remodeling. Histopathology was performed on day 29 post-treatment. Closed arrows, lymphoplasmacellular infiltrates; open arrows, widened alveolar septa; asterisk, bronchus (original magnification, × 10; scale bar, 100 µm). (**G**) Lung collagen content in AdCL (white bars) or AdTGF-β1 (black bars) exposed mice on day 28 after single or repeated LFT, as indicated. Data are presented as mean ± SD of n = 5 mice per group and time point and are representative of two independent experiments. **p < 0.01 compared to disease control (Mann–Whitney U test).

Next, we examined the effect of repeated LFTs on developing lung fibrosis in AdTGF-β1 exposed mice. We first analyzed histological lung sections of AdTGF-β1 or AdCL exposed mice after single or repeated LFT (Fig. 3A–F). Mice exposed to AdTGF-β1 exhibited histopathological features of established lung fibrosis including interstitial collagen deposition along with widened alveolar septa, leading to remodeled lung tissue compared to AdCL treated mice (Fig. 3A–F). Lungs of mice with repeated LFT did not show apparent differences in lung fibrotic remodeling when compared to mice subjected to 1 × LFT on day 28 post AdTGF-β1 (Fig. 3C–F). Similarly, AdTGF-β1 treated mice exhibited no changes in lung histopathology (day 15) after 2 × LFT performed at days 0 and 14 post vector-application (Supplementary Fig. S1).

We next determined collagen contents in lungs of AdTGF-β1 exposed mice subjected to single or repeated LFT. As shown in Fig. 3G, AdTGF-β1 exposed mice exhibited increased lung collagen contents, relative to AdCL treatment on day 28 post-treatment. Again, repeated LFT did not alter lung hydroxyproline contents when compared to single LFT (Fig. 3G), indicating that repeated LFT did not affect developing lung fibrosis in mice.

### Repeated lung function maneuvers do not affect leukocyte subset compositions in lungs of mice

**Figure 4:**
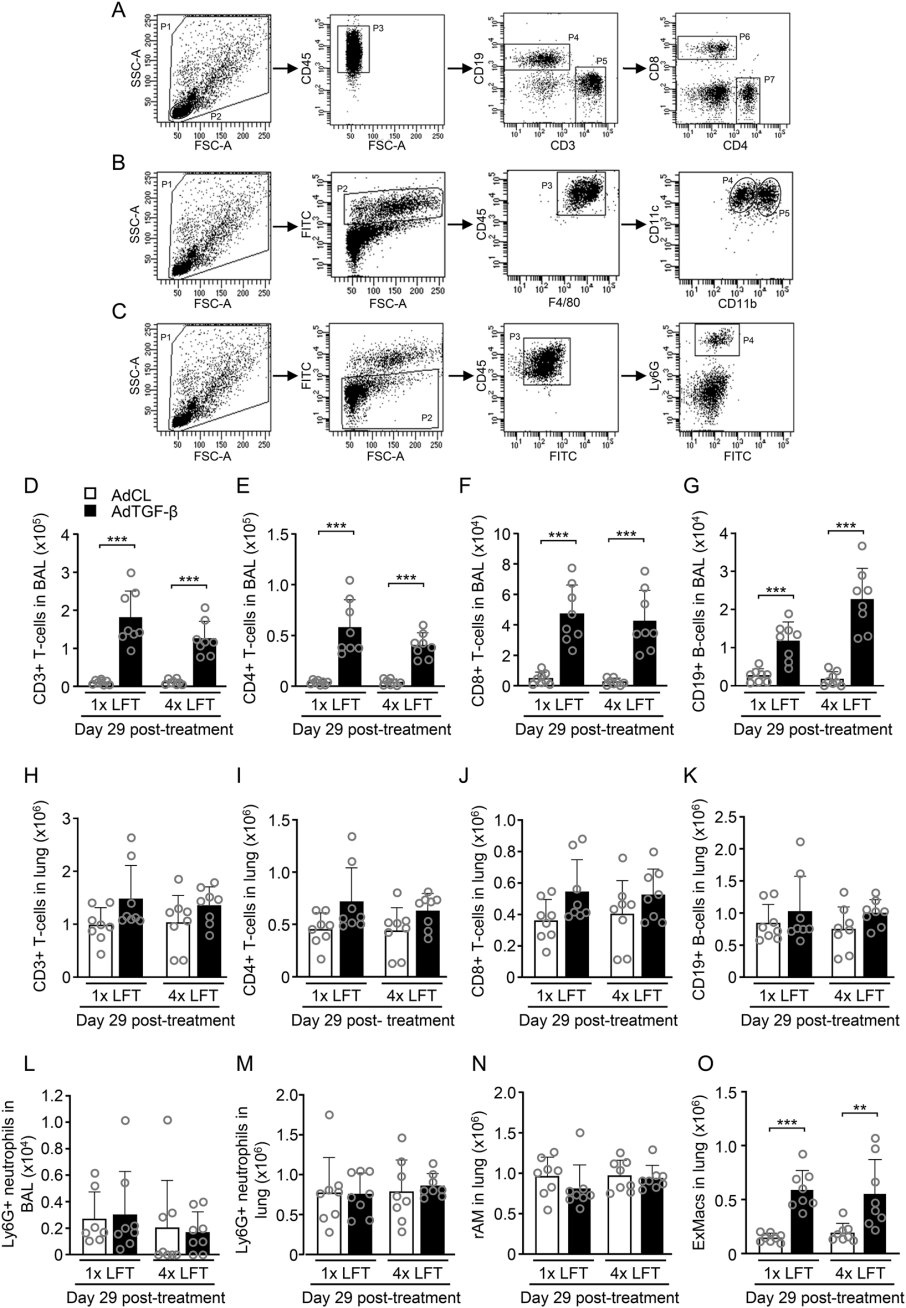
Repeated lung function maneuvers do not affect leukocyte subset compositions in lungs of mice. (**A**–**C**) Immunophenotypic analysis of lymphocytes (**A**), macrophages (**B**) and neutrophils (**C**) in lung parenchyma of AdTGF-β1-exposed mice after 4 × LFT (day 29), as detailed in Materials and Methods. (**D**–**G**) FACS analysis of CD3^+^ T cells (**D**), CD4^+^ T cells (**E**), CD8^+^ T cells (**F**) and CD19^+^ B cells (**G**) in BAL fluid of mice after AdCL (white bar) or AdTGF-β1 (black bar) treatment with single or repeated LFT. (**H**–**K**) FACS analysis of CD3^+^ T cells (**H**), CD4^+^ T cells (**I**), CD8^+^ T cells (**J**) and CD19^+^ B cells (**K**) in lungs of mice after AdCL (white bar) or AdTGF-β1 (black bar) treatment with single or repeated LFT. (**L**–**M**) Neutrophils in BAL (**L**) and lung (**M**) in mice exposed to AdCL (white bar) or AdTGF-β1 (black bar) with single or repeated LFT. (**N**–**O**) Resident alveolar macrophages (rAM; N) and ExMacs (**O**) in mice exposed to AdCL (white bar) or AdTGF-β1 (black bar) with single or repeated LFT, as indicated. Data are shown as mean ± SD of n = 8 mice per time point and experimental group and are representative of two independent experiments. **p < 0.01, ***p < 0.001 compared to disease control or the single LFT group, as indicated (Mann–Whitney U test).

We also questioned whether repeated lung function maneuvers would trigger an influx of inflammatory cells into the lungs of mice. Therefore, we analyzed numbers of macrophages, neutrophils and lymphocytes in BAL and lung tissue of mice by flow cytometry (Fig. 4A–C, Supplementary Fig. S1). As shown in Fig. 4D–F, numbers of CD3^+^/CD4^+^ and C8^+^ T cells were significantly increased in BAL fluids of AdTGF-β1 as compared to AdCL treated mice, both after single and 4 × LFT. Numbers of CD19^+^ B cells were increased in BAL fluids of AdTGF-β1 mice of the 4 × LFT as compared to the 1 × LFT group (Fig. 4G). No significant changes in numbers of CD3^+^/CD4^+^/CD8^+^ T cells or CD19^+^ B cells were seen in lungs of mice treated with AdCL or AdTGF-β1 at day 29 post treatment, and this was independent of whether mice were exposed to single or repeated analysis of lung function (Fig. 4H–K). Numbers of neutrophils in BAL and lung tissue were similar in AdCL or AdTGF-β1 treated mice both after single or repeated LFT (Fig. 4L,M). Also, lung function maneuvers had no effect on numbers of alveolar macrophages in AdTGF-β1 treated mice (rAM, Fig. 4N). Numbers of exudate macrophages (ExMacs, Fig. 4O) increased in lungs of AdTGF-β1 treated mice, but no effect of repeated LFT on numbers of ExMacs was observed in mice with established lung fibrosis. In addition, no effect on distribution of BAL cell constituents was observed in AdTGF-β1 mice subjected to 2 × LFT compared to 0 × LFT (Supplementary Fig. S1). Together, these data show that repeated LFT did not alter the pattern of BAL leukocyte accumulations in mice exposed to control vector or AdTGF-β1.

## Discussion

In this study we questioned whether repeated LFT would be feasible in healthy mice and mice with experimental lung fibrosis. Using flexiVent based lung function tests in conjunction with flow cytometry analyses we show that repeated LFT does not trigger overt inflammation in healthy or pre-injured lungs or worsen lung fibrosis and has no impact on overall well-being of the mice.

Animal models play an important role in pulmonary research. Lung function measurement is critical for the diagnosis and monitoring of chronic lung diseases, such as pulmonary fibrosis. Different to humans, assessment of lung function in mice is usually performed as a single endpoint measurement, thereby allowing evaluation of the functional status at one defined time point only. Here we challenged healthy mice and mice with lung fibrosis with four lung function maneuvers separated by 7 and 14 days, respectively between measurements. Repetitive assessment of lung function at 0–14–21–28 days was well tolerated in control mice and mice with lung fibrosis. This study excludes in a preclinical model system an effect of repeated lung function assessment on developing lung fibrosis in mice, which may be of clinical interest, even though invasive lung function maneuvers in mice cannot be directly compared to non-invasive pulmonary function tests in patients with developing fibrosis.

In general, mice tolerated repetitive intubation and LFTs well. A small decrease in body weight was noted at 24 h post LFT, which normalized within 72 h. The observed drop in body weight occurred in both experimental arms, i.e. mice with single or repetitive lung function maneuvers with or without underlying fibrosis. Therefore, the observed weight loss was most likely an anesthesia- rather than lung function-related effect. Our data are consistent with previous studies also demonstrating weight loss after repetitive LFT -albeit reported in healthy mice- which normalized after 7–12 days^16,17^.

In the current study, we used female mice mainly because of housing reasons. Some studies reported sex-dependent differences in LFT parameters in the bleomycin model of lung fibrosis^25,26^. However, in a previous study of our group, we did not observe any differences in lung function in AdTGF-β1 treated male and female mice after a single LFT^22^, suggesting no impact of sex in the currently employed fibrosis model.

Previous studies reported the feasibility of repeated LFTs in healthy mice at two or three consecutive time points^16,17^. Our study is the first to address the effect of four consecutive LFTs on lung micromechanics, lung histopathology and biochemical parameters in individual healthy and diseased mice over a period of 28 days. We observed that this experimental maneuver was well tolerated and did not affect developing lung fibrosis in mice. Thus, repeated LFT is feasible in the AdTGF-β1 mouse model, and thus allows a better understanding of disease development in preclinical model systems.

Catheterization of mice for invasive lung function assessments is an important issue since even small “gaps” between the trachea and the catheter may cause pressure drops and leakage and may promote spontaneous breathing. Others used 18 G catheters to avoid leakage and pressure drops during ventilation and respiratory maneuvers^16,17^. Here we employed 19 G catheters for intubation, and we did not encounter any problems with pressure drops or spontaneous breathing of mice, which would have been displayed by the flexiVent system with parameter values far beyond standard. Our choice of 19G catheters was mainly attributed to the finding that these catheters caused no injury to the inner surface of the trachea during intubation.

Fibrotic remodeling of lung tissue causes lung stiffness and impaired gas exchange, eventually leading to respiratory failure^3,27^. Mechanical ventilation of an injured lung may also promote lung fibrosis^28–30^. In line with this, previous studies showed that mechanical ventilation did not improve outcome of IPF patients with acute respiratory failure^31,32^, as excess stretch and strain during ventilation may trigger increased profibrotic responses^28,33,34^. Both LFT and mechanical ventilation impose mechanical forces to the lung tissue, albeit at different levels and durations. In this study, exposure of mice with developing lung fibrosis to four times LFT had no impact on developing lung fibrosis, and as such is feasible and well tolerated even in mice with pre-injured lungs.

Previous studies reported increased neutrophil counts in BAL after repetitive lung function measurement in intubated as compared to tracheotomized mice, although histological sections of lungs appeared normal without signs of inflammation^16^. In our studies, we did not observe increased lymphocyte or neutrophil counts in BAL or lung tissue after four lung function maneuvers, and proinflammatory cytokines like TNFα and Il-1β were not detectable in BAL fluids of AdTGF-β1 exposed mice after one or four LFTs. Based on these data, repeated LFT does not seem to have a proinflammatory effect in healthy mice or mice with developing lung fibrosis.

Repeated measurement of lung function in the same mouse has several advantages: (i) longitudinal monitoring of disease development within an individual, (ii) reduced numbers of mice per experiment, thereby supporting the 3R principle, (iii) reduced animal costs per experimental study. Clearly, from an ethical perspective, the slightly higher experimental burden for fewer mice appears justified in view of the considerable reduction of numbers of mice otherwise needed for lung function studies.

Together, our data show that repeated lung function maneuvers in the same mice with developing lung fibrosis were feasible and well tolerated, reduced animal numbers per experiment and allowed a detailed longitudinal evaluation of lung mechanics. In addition, repeated lung function measurement had no impact on lung leukocytic responses nor did it affect developing lung fibrosis in mice.

### Supplementary Information

Supplementary Figure S1.

## Data Availability

All the data contained within the manuscript will be made available from the corresponding author upon reasonable request.
