# Supplementary material for: Repetitive invasive lung function maneuvers do not accentuate experimental fibrosis in mice
Source: Sci Rep. 2024 Jun 14;14:13774. doi: 10.1038/s41598-024-64548-w (PMC11178923; doi:10.1038/s41598-024-64548-w)
Supplement: Supplementary file 1 — Supplementary Figure S1. [file 41598_2024_64548_MOESM1_ESM.pdf]

Supplementary Information

Repetitive invasive lung function maneuvers do not accentuate experimental fibrosis in mice

Tina Röpke, Franziska Aschenbrenner, Lars Knudsen, Tobias Welte, Martin Kolb, and Ulrich A. Maus

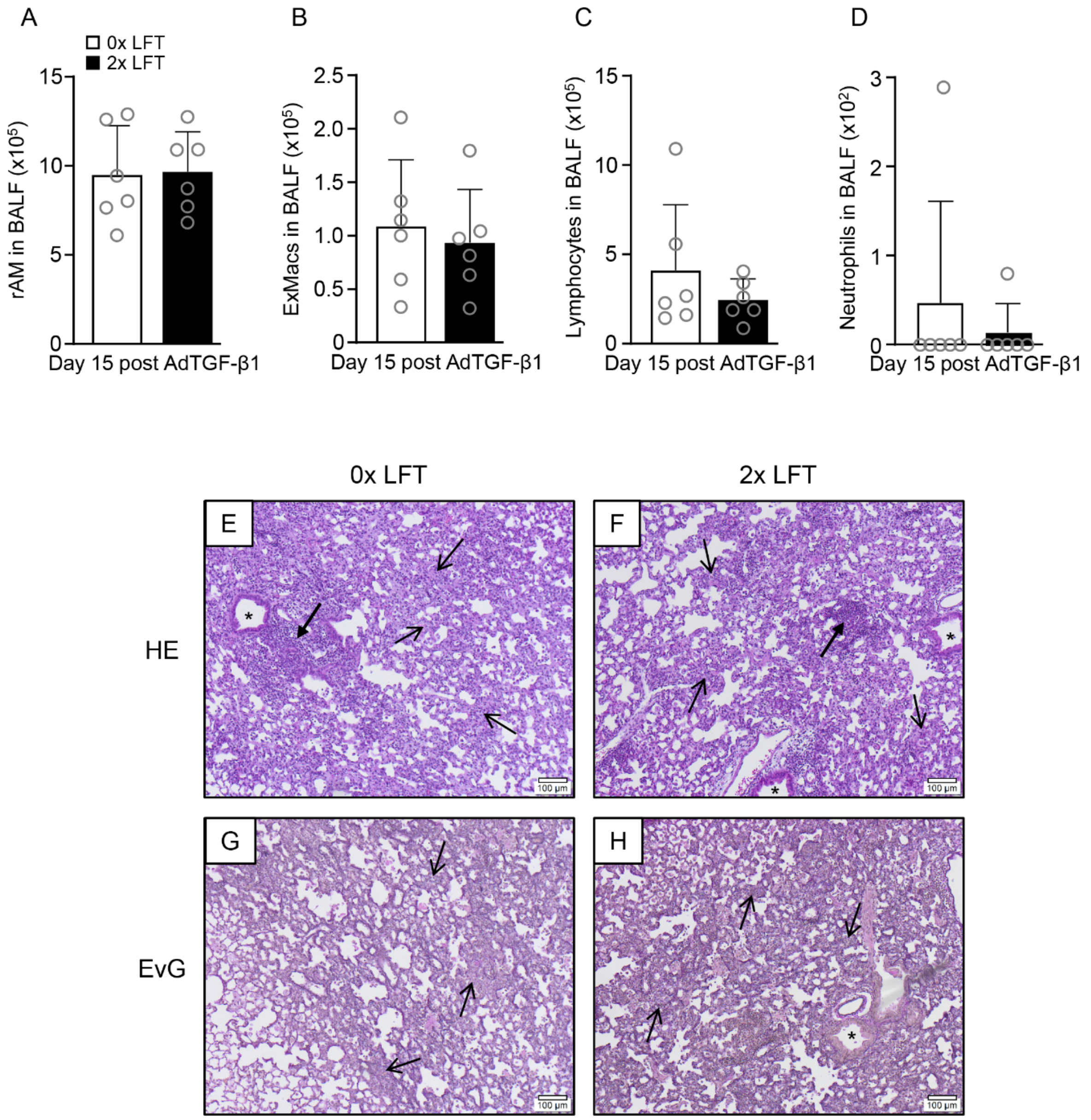

Figure S1. Repeated lung function maneuvers do not affect cellular composition in BAL or histopathology of mice with lung fibrosis at day 15

(A-D) Resident alveolar macrophages (rAM; A), exudate macrophages (ExMacs; B), lymphocytes (C) and neutrophils (D) in BAL fluid of mice exposed to AdTGF-β1 without LFT (0x LFT, white bars) or after two LFT (2x LFT, black bars) at day 0 and 14, as indicated. (E-H) Histopathology of mice exposed to AdTGF-β1 without LFT (0x LFT) (E,G) or after two LFT (2x LFT) (F,H) at day 0 and 14. Lung sections were stained with hematoxylin/eosin (HE) (E,F) or Elastica van Gieson (EvG) (G,H) for assessment of lung tissue remodeling. Histopathology was performed on day 15 post-treatment. Closed arrows, lymphoplasmacellular infiltrates; open arrows, widened alveolar septa; asterisk, bronchus (original magnification, x 10; scale bar, 100 µm). Data are shown as mean ± SD of n = 6 mice per time point and experimental group.
